# Online gallery facilitated art activities for people with dementia during the COVID-19 pandemic and beyond: A narrative review

**DOI:** 10.1177/14713012231198748

**Published:** 2023-08-30

**Authors:** Lara Wiseman, Stephen Isbel, Adriane Boag, Carolyn Halpin-Healy, Diane Gibson, Kasia Bail, James M Noble, Nathan M D’Cunha

**Affiliations:** Faculty of Health, 110446 University of Canberra, Bruce, ACT, Australia; Ageing Research Group, Faculty of Health, 110446 University of Canberra, Bruce, ACT, Australia; 2218 National Gallery of Australia, Parkes, ACT, Australia; Arts and Minds, New York, NY, USA; Faculty of Health, 110446 University of Canberra, Bruce, ACT, Australia; Ageing Research Group, Faculty of Health, 110446 University of Canberra, Bruce, ACT, Australia; Arts and Minds, New York, NY, USA; Taub Institute, 21611 Columbia University Irving Medical Center, New York, NY, USA; Faculty of Health, 110446 University of Canberra, Bruce, ACT, Australia; Ageing Research Group, Faculty of Health, 110446 University of Canberra, Bruce, ACT, Australia

**Keywords:** art gallery, technology, dementia, Alzheimer’s disease, museum

## Abstract

Art activities for people with dementia have a range of therapeutic benefits including psychosocial wellbeing and enhanced quality of life. Successful art programs promote social engagement, are inclusive and empowering, and enable opportunity for people with dementia to express themselves verbally and non-verbally. The COVID-19 pandemic and associated social distancing precautions have impacted the capacity of art galleries and museums to deliver in-person programs. However, they have also provided a new opportunity. This paper explores the potential benefits, challenges, and future directions for research relating to the online delivery of gallery-facilitated art activities for people with dementia. The evidence revealed that increased digitisation of programs increased access for participants, however, the majority of the research was published before the pandemic. Nevertheless, COVID-19 has necessitated many museums and galleries to engage with people with dementia online. Future research is needed to improve the usability of online delivery platforms and a comparison of online and onsite delivery is recommended, particularly to evaluate benefits to people living in rural and remote areas where access to museums and galleries may be limited.

## Introduction

Over the past two decades, art galleries and museums around the world have developed access programs to provide art activities for people with dementia. Often developed in partnership with health, dementia support or aged care services, these access programs have the potential to enhance the psychosocial and psychophysiological wellbeing of people with dementia, providing an opportunity for intellectual stimulation, creative engagement, social interaction, and personal reflection (Camic et al., 2016; Cavalcanti Barroso et al., 2022; D'Cunha et al., 2020; Ullán et al., 2013). A taxonomy of arts interventions for people with dementia listed eight principles and features: transformation, possibility, connection, expression, involvement, selfhood, humanity, and engagement (Cousins et al., 2020). Connecting with art in galleries and museums also has the potential to provide new perspectives on life, while activating emotional and bodily responses of people with dementia (Lea & Synnes, 2021). Importantly, art and dementia programs featured in museums and galleries are inclusive and empowering, pushing back against fear and stigma in a spirit of solidarity (Halpin-Healy, 2017).

People with dementia have long been part of art gallery and museum audiences. Following from early evaluations demonstrating people with dementia benefitted from painting as part of the Memories in the Making art program in Cincinnati (Kinney & Rentz, 2005; Rentz, 2002), the Museum of Modern Art (MoMA) became the first art gallery to formalise dementia-focused programs and evaluate their impact in New York in the 2000s (Rosenberg, 2009; Rosenberg et al., 2009). The Meet Me at MoMA program for people with dementia and their carers involves interactive tours of the gallery collection. Subsequently, many art galleries and museums throughout the United States (Burnside et al., 2017; Halpin-Healy, 2017; Halpin-Healy & Noble, 2020; Lamar & Luke, 2016; Rhoads, 2009) and internationally, including Australia (Boag, 2014; Heldon et al., 2020; MacPherson et al., 2009), Canada (Hazzan et al., 2016; Humphrey et al., 2019), the Netherlands (Hendriks et al., 2019), Germany (Schall et al., 2018) and the United Kingdom (Camic et al., 2014) have developed their own access programs for people with dementia.

There are generally two elements to these gallery or museum-based access programs: art conversations which involve viewing and discussing paintings, photographs, sculptures, textiles, ceramics, glass, and other objects within a group setting (MacPherson et al., 2009); and/or art-making sessions inspired by works of art from the galleries’ collections involving a range of activities such as painting, drawing, working with clay, printmaking, collage, poetry and story building (Camic et al., 2014; Clarke-Vivier et al., 2017; Cousins et al., 2020; Eekelaar et al., 2012; Halpin-Healy, 2015; Heldon et al., 2020; Rosenberg et al., 2009). Participants may be involved in one or both elements of the programs (Shoesmith et al., 2020). The programs are facilitated as group activities, with social engagement being a key feature (Bourne et al., 2021), and may be attended individually by people with dementia or by dyads of people with dementia and a family member or care partner (Bourne et al., 2021; Burnside et al., 2017; Camic et al., 2014, 2016; Eekelaar et al., 2012; Flatt et al., 2015; Hazzan et al., 2016; MacPherson et al., 2009; Rosenberg, 2009).

One of the characteristics that distinguish these programs from other art interventions or therapies for people with dementia is that these sessions are conducted within gallery or museum settings and are facilitated by gallery staff who are experienced museum educators who have also received dementia-specific training (Boag, 2014; Flatt et al., 2015; Halpin-Healy, 2017; MacPherson et al., 2009). Windle et al. (2018) identified that skilled facilitators see the potential of what can be achieved by the person with dementia, understand and allow for individual needs and abilities, and offer guidance and support when needed. Boag (2014) describes how facilitators involved in the delivery of such programs closely observe participants, actively listen, use non-verbal communication cues and silence, to give participants time to observe the works of art, process information and contribute to discussion. The staff involved in delivery of these gallery or museum-based programs commonly use Visual Thinking Strategies, an inquiry-based approach providing a safe and supportive environment where people with dementia are valued for their contributions (Burnside et al., 2017; Lamar & Luke, 2016). Shoesmith et al. (2020) suggest that the combination of artistic skill, art historical knowledge, and dementia awareness is an important aspect of providing skilled facilitation along with person-centred core values such as compassion, empathy and patience. Burnside et al. (2017) reporting on qualitative findings from dyads involved in the *here:now* program at the Frye Art Museum, identified how the skills of the facilitator influence participation and enjoyment of art access programs.

A decade ago, Beard (2012) identified that within the literature relating to art and dementia, there is a distinction to be made between art therapy (a form of psychotherapy that generally occurs in clinical settings and is delivered by licensed therapists) and art activities (with a focus on engaging in art or with the arts as a creative, social and leisure activity). Following this distinction, these access programs are art activities rather than art therapy, although such programs may have a range of therapeutic benefits (Cavalcanti Barroso et al., 2022; D’Cunha et al., 2019; Young et al., 2015). Ward et al. (2021) describe such activities as participatory art activities that take place in community settings and are delivered by art professionals. Clarke-Vivier et al. (2017) report how the development of an art-making activity to complement an existing art conversation program, sought to provide participants with an opportunity to use art as a way to express themselves creatively, engage in different forms of communication and experience success in art making, to promote elements of wellbeing such as social engagement, self-expression and positive mood.

Cotter and Pawelski (2022) examined the unique role that galleries and museums may play in enhancing the wellbeing of people with dementia (and others) as institutions of human flourishing, highlighting the impact of activities held within gallery settings. Camic et al. (2016, p. 11) observed that the experience of being in the gallery environment provides people with dementia with the opportunity to enjoy an intellectually stimulating activity in a “special and valued place.” Qualitative research by Burnside et al. (2017) also highlighted the importance of the gallery setting for facilitating engagement. The growing recognition of the positive impact of such programs on health and wellbeing of people with dementia (and other groups) has prompted the introduction of social prescribing, whereby people are referred by doctors or other health professionals to arts activities within their community to increase social engagement and participation in positive health-related behaviours (All-Party Parliamentary Group on Arts, 2017; Camic & Chatterjee, 2013; Cotter & Pawelski, 2022; Davies et al., 2016). This intersection between the arts and health is further reflected in the research collaborations between galleries, museums and health academics involved in dementia-related research (Camic et al., 2014; D’Cunha et al., 2019; Flatt et al., 2015; Hazzan et al., 2016; Humphrey et al., 2019; Schall et al., 2018).

While there is a growing body of literature describing gallery-based (and other) art activities for people with dementia, the evidence relating to effects requires further scrutiny. For example, a review by Cavalcanti Barroso et al. (2022) on participatory visual art-making activities (delivered in gallery and other settings) highlighted that while numerous studies have shown benefits for participants, there are inconsistencies in the literature due to diversity in study design and the format of the delivery of the programs. Similarly, a systematic review undertaken by Bourne et al. (2021) reporting on psychosocial outcomes of dyadic arts interventions (including art viewing and art-making) for people with dementia and their informal caregivers, concluded that despite generally positive findings, it was difficult to make comparisons across the different interventions due to small sample sizes, lack of control groups and the use of different outcome measures. Bourne et al. (2021) concluded that further research with comparable theoretically informed and dementia-specific outcome measures is necessary. Manera et al. (2022) used a Delphi methodology to develop recommendations for the structure, format and content of workshops both in-person and online (involving arts and games) for people with neurocognitive disorders (including dementia) in an effort to increase standardisation of procedures used in practice and research.

Schneider (2018) elaborated on the relevance of context, arguing that evaluation of the impact of the intervention requires consideration of the intervention level, the degree of participant’s cognitive impairment (stage of dementia), and who delivers the intervention. The intervention level refers to whether the program targets an individual (the person with dementia), a dyad (person with dementia and their carer), or a broader group, such as within a residential aged care setting (Schneider, 2018). Humphrey et al. (2019) identify three “pillars” in their framework for successful engagement: a dementia-friendly environment; supportive communication strategies (approach); and a suitable, well-planned activity providing descriptions of how each of these may be achieved. The level of cognitive impairment of participants is important to better understand what types of interventions work best at which stages of the disease, with which outcomes, and to document the appropriateness of different evaluation methods (Cousins et al., 2020). Finally, the skills, qualifications, knowledge and experience of the person delivering the program will affect all aspects of program delivery; a point further supported in a systematic review by Shoesmith et al. (2020).

Other variations in the delivery and evaluation of gallery-based art activities include the frequency and duration of the individual sessions, as well as the duration of the program (ongoing, short-term or single intervention); the number of participants, the type of art-making activities, the types of outcomes that are measured and the evaluation tools used. In describing the elements required to create an effective visual art intervention for people with dementia, Shoesmith et al. (2020) found that in terms of frequency, weekly sessions were most likely to lead to positive outcomes and recommend the duration of the session (usually ranging from 60 to 120 min) should be adapted to suit the capacity of participants and that opportunity for social interaction provided by these activities was as important as the art activities. Across the 21 included papers, the three main outcomes reported were: social inclusion and connectedness, wellbeing and cognitive stimulation (Shoesmith et al., 2020). The importance of meaningful social interaction was further supported in a qualitative study by the same researchers (Shoesmith et al., 2021).

Prior to the acute respiratory syndrome coronavirus 2 (COVID-19) pandemic, such programs involved participants visiting the gallery or museum to share the experience of art conversations and/or art-making activities together with an arts educator/facilitator and other individuals living with dementia or dyads. However, as galleries and museums worldwide closed their doors due to stay-at-home directives implemented to limit the transmission of COVID-19, the continuation of such programs required a transition from onsite to online delivery (Boag et al., 2022). While opportunities for onsite programs have resumed in many places, some galleries continue offering online sessions as part of their access programs for people with dementia. Such programs offer new ways for people with dementia to engage with art and provide galleries with new opportunities to extend and increase their outreach to this population group.

During the COVID-19 pandemic, many cultural institutions implemented or expanded digital access to their collections to sustain and develop programs already in place and provide a potential hybrid model for future delivery. However, there is concern that these technologies introduce new barriers for those with limited resources, computing or otherwise. This review sought to identify and synthesise the current literature relating to the online delivery of gallery-facilitated art discussion groups and art-making groups for people with dementia. In addition, we explore how digital technology opportunities compensated for COVID restrictions, highlighting the unexamined emergence and evolution of art activities facilitated online since 2020 in response to the COVID-19 pandemic. Accordingly, this review draws on the larger body of literature relating to gallery-facilitated onsite art activities and the use of everyday technology by people with dementia to participate in socially engaging and interactive art or other creative activities.

The aim of the review is to identify potential benefits, opportunities and challenges to implementing online gallery-facilitated art activities for people with dementia.

## Methods

In 2022, a non-systematic literature search was performed using the Web of Science, PubMed, and CINAHL electronic databases to identify full-text journal articles published in English, using the following search terms: “art” and “dementia OR Alzheimer’s” and “gallery OR museum.” This yielded approximately 200 relevant articles. The search was extended to include the first 25 pages (250 entries) of Google Scholar. A review of reference lists of relevant articles identified during the searches was also undertaken and included grey literature reports and publications. The primary aim was to identify peer-reviewed articles published since 2005 (reflecting commencement of the highly influential gallery-facilitated art program for people with dementia at the New York Museum of Modern Art (Rosenberg, 2009)), to determine if such programs were being delivered online prior to and during the COVID-19 pandemic. This search strategy yielded information about the use of digital technology for viewing art, but we did not find evidence of the delivery of gallery-facilitated art viewing or art-making programs for people with dementia via online video conferencing platforms prior to 2020, indicating that the COVID-19 pandemic served as the catalyst for the introduction of such programs. Due to the paucity of academic research relating to this specific type of activity for people with dementia, the available information relating to the delivery of online facilitated arts-based activities for people with dementia was reviewed to identify issues that may be relevant to the delivery of gallery-facilitated art conversation or art-making activity for either people with dementia and/or carers using technology remotely. Due to the recent commencement of these programs, a general internet search of art galleries and museums was also conducted to identify online programs delivered in response to pandemic-related disruptions. Human ethics approval was not required for this review.

## Results

The following synthesis provides descriptors of current literature and emerging evidence of the state and role of digital technology in supporting people with dementia to inform ongoing developments. In total, 198 articles were identified through the database search, and 448 including Google Scholar. Of these, 34 are included in the results discussion below, 27 were published between 2020 and 2022, and only five peer-reviewed articles relating specifically to the COVID-19 pandemic were identified (see Table 1). Four themes emerged from the overall literature synthesis: (1) the role of digital technology in mitigating the impact of COVID-19 on people with dementia and their carers; (2) the impact of the COVID-19 pandemic on gallery-based art activities for people with dementia; (3) the use of everyday technologies by people with dementia to engage in art activities; and (4) the way in which the COVID-19 pandemic served as a catalyst for increased technology use in older people.

**Table 1.** Summary of literature of art activity interventions and studies during the COVID-19 pandemic.

Author, Country	Study design	Participants	Outcome measures	Results
Brandão et al. (2022), Brazil	Mixed-methods feasibility study of a remote intergenerational intervention to promote wellbeing and social connection in vulnerable older people called *Playful Living*. With guidance, 20 undergraduate students from Speech Language Therapy, Drama, Psychology, Dance, Gastronomy, Psychiatry and Public Health facilitated group sessions of art-related activities: clowning, storytelling, dancing and cooking in groups of 7–8 older people. Length: Weekly 60 min sessions for 3 months. Delivery: Zoom (group sessions), WhatsApp (1:1 video calls at least 1/week student & participant).	*n* = 24 People aged 60 or over with stroke-induced cognitive expressive aphasia (*n* = 13), stroke-induced cognitive complaints (*n* = 2), people with mild to moderate dementia (*n* = 5), and people without any neurological conditions (*n* = 4).	Quantitative: Participation and attendance. Qualitative: Participative observation and thematic analysis of evaluation conversations.	Attendance: 67% had high attendance (9–12 meetings). Participatory observations: Structure of sessions and activities were well received. Evaluative conversations: Program perceived as supportive and reported feelings of belonging, positive comments relating to social connection, engagement and learning from each other (students and participants).
Lee et al. (2021), Ireland	Qualitative evaluation of the facilitation of dementia-inclusive online singing groups. Previous on-site singing group program for community-dwelling people with dementia transitioned to online delivery during COVID-19 restrictions. Option of engaging in one or more online musical activities (WhatsApp singalongs, Facebook public live stream, YouTube playlists and singalongs, virtual choir recordings, music appreciation). Length: 6 × online singing sessions/choir rehearsals. Delivery: Zoom, WhatsApp, YouTube, Facebook.	*n* = 12 Participants were facilitators of dementia-inclusive singing groups or choirs in Ireland.	Qualitative: Thematic analysis of semi-structured interviews with facilitators of dementia-inclusive singing groups or choirs were conducted (September to October 2020 via voice calls).	Four themes: 1. Accessibility of online delivery: provided capacity to reach people who might not be able to attend in-person, but also potential for technology to create barriers to participation. 2. Online versus in-person delivery: recognition that singing together in person is a unique experience, but positive experience for those engaged in online activity when in-person not an option. 3. Importance of social connection: social connection can occur in online environment between facilitators and group members. 4. Adaptability and resilience: people with dementia engaged with technology and participated, and facilitators adapted to online delivery challenges.
Quail et al. (2021), United Kingdom	Mixed-methods case study of a multimodal intervention (previously delivered by trained therapists in-person) to individuals and groups in communities in the UK and China to online delivery during COVID-19 (UK). 1:1 video or telephone consultations with therapists who scripted and filmed a series of therapeutic activities and explanatory videos. Duration/Length: Online delivery April -May 2020 × weekly 30-min sessions increasing to 3× weekly 30-min sessions (June – July 2020) Delivery: Skype, YouTube	*n* = 1 Male in late 60’s with Alzheimer’s disease with spouse as carergiver.	Quantitative: Cessation of scored assessments during online program due to lack of validation for online use of the dementia assessment scoring tools. Qualitative: Observations of on-screen interaction (observed changes in mood, functioning & cognition).	Carer supervision/support required for online session duration (compared to opportunity for respite during in-person sessions) and type of activities used were limited by digital delivery. There were safety-netting concerns as therapist not physically present. Maintaining meaningful engagement through online sessions is important to mitigate against deterioration in cognition resulting from interruption to in-person therapeutic programs. Online sessions were a positive experience for participant and carer (improvement in mood and shared activities to discuss).
Stewart et al. (2022), Scotland	Mixed methods study aimed at supporting wellbeing of people with dementia and their caregivers through online cultural engagement events set up in response to COVID-19. 30 online group sessions tailored to people with dementia including: Two activities: GLAM (Galleries, Gardens, Libraries, Art, Museums and Music) and Dementia Socials. Involved seven Cultural and heritage organisations. Duration/Length: Each institution hosted three events, one per month for 3 months. Delivery: Zoom.	Number of participants was not specified for each session but included between 12 and 20 participants (people with younger-onset dementia, carers, family members, and people with other neurodegenerative conditions) per session.	Quantitative: Observations and attendance. Qualitative: Online focus groups (5 with 20 participants each including people with dementia and carers), semi-structured interviews using Microsoft Teams, interviews with each of the eight event organisers and one dementia training coordinator.	Online events provided opportunity to connect communities from remote and international locations through participating in online group activities from home. This promoted engagement and boosted sense of wellbeing. Recommendations were developed relating to outreach and engagement, access to appropriate event information, including opportunities for participant-led discussions, regularly scheduled events, and combining of online and on-site delivery.
Talbot and Briggs (2022), United Kingdom	Qualitative study of the use of digital technologies by people with dementia during COVID-19. Participants reported involvement in a wide range of activities including: video calls, attending online dementia groups, online shopping, exercises classes, bird-watching, theatre performances, photo and music discussions, and religious services. Delivery methods mentioned: Zoom, YouTube, Twitter, Microsoft.	*n* = 19 People with mild to moderate dementia aged between 50 and 84 years, including 14 with younger onset dementia.	Qualitative: Interviews via video-calling software (*n* = 9), telephone (*n* = 9) or email (*n* = 1).	Five themes: 1. Technologies to assist with everyday life. 2. Technologies for social connection. 3. Technologies to feel good. 4. Technologies to find meaning. 5. Hypercognitive technologies. Overall findings related to using video-calling for social engagement & connecting with peers (through online participation in dementia & activity groups). Challenges included need for training on use of digital technology, navigating digital technology use, need for dementia-specific accessibility resources, and cognitive (Zoom) fatigue.

### Role of digital technology in mitigating the impact of COVID-19 on people with dementia and their carers

The findings of fifteen articles were relevant to how digital technology may mitigate the impact of COVID-19 restrictions (Bacsu et al., 2021; Boots et al., 2014; Brandão et al., 2022; Chu et al., 2021; Cuffaro et al., 2020; Curelaru et al., 2021; Heins et al., 2021; Howard et al., 2021; Hung et al., 2018, 2019, 2021; O’Rourke et al., 2021; Quail et al., 2021; Tuijt et al., 2021; Salva et al., 2020). The COVID-19 pandemic has and continues to seriously impact the morbidity and mortality of people with dementia (Bacsu et al., 2021). While many of the protective measures introduced by governments in response to the COVID-19 pandemic have sought to limit the transmission of the disease, particularly among vulnerable members of society, these same restrictions have also meant that many people with dementia (like many other citizens) were not able to maintain their daily routines, experienced increased social isolation and reduced access to social and other support services (Brandão et al., 2022; Curelaru et al., 2021; Heins et al., 2021; O'Rourke et al., 2021; Quail et al., 2021). In particular, people living in residential aged care had limited access to outdoor physical activities. Accordingly, the ability to participate in many activities they previously enjoyed was hindered during the COVID-19 pandemic heightening the risk of social isolation (Curelaru et al., 2021; Tuijt et al., 2021).

Quail et al. (2021) argue that continuity of care is important and that remote digital technology can be used to counteract the potentially negative impact of interruptions on providing in-person support, therapeutic programs, meaningful activities, and social engagement. A qualitative study conducted in London in 2020 by Tuijt et al. (2021) indicated that people with dementia found it difficult to replace their regular activities involving social engagement, as access to activities such as memory cafés, exercise groups and day centres was restricted and that those who could adapt old or create new routines within their home environment appeared better able to cope during the lockdown. Another study conducted in England by O’Rourke et al. (2021) found that a move to the online delivery of some activities, such as religious services or memory cafés, compensated partly for the physical closure of many community venues. People with dementia and carers reported using various video conferencing platforms in an effort to provide opportunities for social engagement, however study participants noted that technological barriers and digital exclusion could affect the extent to which such an approach could be implemented (O'Rourke et al., 2021; Tuijt et al., 2021). Heins et al. (2021) reviewed the effects of technological interventions on social participation of community-dwelling older people with and without dementia. The majority of identified studies examined the use of communicating on the internet, finding only limited effects on loneliness, social support, and social isolation, and face-to-face contact via electronic means was suggested as key element of successful technological interventions. The authors (Heins et al., 2021) concluded that more studies are needed to examine using technology to facilitate participation in out-of-home social activities.

In addition to people with dementia, carers were also impacted by stay-at-home restrictions imposed in many countries during the pandemic. The suspension of activities in community settings reduced the opportunities for physical and psychological respite that many carers rely on in order to manage their own self-care, work and participate in other activities (O'Rourke et al., 2021). Telephone interviews with carers of family members with dementia in rural Virginia (USA) conducted by Savla et al. (2020) during the early stages of the pandemic found that while many reported negative impacts relating to high role overload (47%) and insufficient family support (32%) as family members were unable to visit, a small minority (11.3%) identified some positive impacts. For example, changes to employment circumstances enabled them to spend more time with their family member with dementia, or get assistance from other family members who would usually not have been available (Savla et al., 2020). While this study was not examining art interventions, the authors noted that access to internet-based services is particularly convenient for people in rural areas who might otherwise have to travel long distances to access various services (Savla et al., 2020). An earlier systematic review found that carers of people with dementia benefit from online support programs that provide a mix of information, customised caregiving strategies, and contact with other carers (Boots et al., 2014).

The restrictions imposed in response to COVID-19 accelerated the use of digital technologies, including video conferencing for telemedicine and telehealth services for dementia care (Cuffaro et al., 2020). Digital delivery of such services has helped improve access to a wide range of dementia support services, particularly for those with mobility and/or transport issues, immunocompromised, and those located in regional or remote areas. However, access relies on having fast and reliable internet services and the related technological devices used to access internet-based communication tools (Cuffaro et al., 2020). For many people with dementia living in residential aged care homes, their capacity to access such services was and, in many cases, continues to be limited by the lack of information and communication technology infrastructure to enable residents to engage in internet-based activities (Chu et al., 2021). An innovative pre-COVID-19 exception to this is the *ArtontheBrain* program, a web-based program offering people with dementia in long term care the opportunity to engage in three different types of art activities on an iPad (Howard et al., 2021).

### The impact of the COVID-19 pandemic on gallery-based art activities for people with dementia

Nine articles described the impact of COVID-19 on engaging with gallery-based art activities (Bascu et al., 2021; Bradbury et al., 2021; Czech et al., 2022; Grácio, 2020; Holcombe-James, 2021; Howard et al., 2021; International Council of Museums, 2020; Stewart et al., 2022; Talbot & Briggs, 2022). The COVID-19 pandemic prompted many governments to impose a range of restrictions, including social/physical distancing and lockdown/stay-at-home measures designed to curb the spread of COVID-19. These restrictions extended to the closure of non-essential businesses forcing most art galleries around the world to close their doors to visitors for several months during 2020 and 2021 (Bradbury et al., 2021; Holcombe-James, 2021). The widespread closure of public institutions necessitated a rapid transition to digital delivery of art programs (Holcombe-James, 2021; International Council of Museums, 2020). The capacity of art galleries (and other cultural institutions) to provide digital access to their collections via online platforms during the COVID-19 pandemic closures varied enormously and relied heavily on the level of digital investment made by institutions prior to 2020 (Grácio, 2020; Holcombe-James, 2021). For art galleries that had made this investment prior to the pandemic, closures provided new opportunities to expand the delivery of their art access activities. However, one of the unknown elements of this transition was whether people with dementia who had previously participated in onsite programs would have access to the necessary equipment and support to participate in online art activities and whether they would be interested in doing so.

Although not specific to gallery-based art conversations or art making, an action research project by Stewart et al. (2022) identified challenges in adapting cultural events from an onsite to online format to facilitate maximum engagement and participation by attendees. The authors also found that online events provide an opportunity for social connection and stimulation for individuals unable to attend onsite events due to COVID-19 restrictions, but also due to mobility and/or transportation issues; that participation in online group activity from the home environment encourages attendees’ engagement and boosts their sense of well-being; and weekly participation provides structure, connection and builds self-confidence (Stewart et al., 2022). In addition, Czech et al. (2022) discuss how organisational interdependencies such as finances and networks determined what online dementia-friendly activities could be delivered. Boag et al. (2022) similarly highlight how the transition to the delivery of online programs was supported by an international network of gallery staff involved in a community of practice to deliver art and dementia programs. While the published evidence is limited, there are emerging reports of positive adaptations during the pandemic that reduce social isolation for people with dementia, including online art activities (Czech et al., 2022; Stewart et al., 2022) and online support groups (Talbot & Briggs, 2022).

### The use of everyday technologies by people with dementia to engage in gallery-based art activities

We identified 11 articles providing insights on how everyday technologies could provide a medium for engaging in gallery-based art activities during a pandemic (Boag et al., 2022; Bradbury et al., 2021; Evans et al., 2015; Hung et al., 2018, 2019, 2021; Joddrell & Smith, 2019; MacRitchie et al., 2022; Stewart et al., 2022; Sweeney et al., 2021; Tyack & Camic, 2017; Tyack et al., 2017). Jodderell and Smith (2019) provide an overview of the importance of enabling people with dementia to continue engaging in social and leisure activities and how technology may be incorporated into people’s lives. The authors describe underutilisation of currently available technologies to support people with dementia, and the potential benefits to carers for access to peer support, problem solving, and information. A systematic review of dementia-focused assistive technology conducted by Evans et al. (2015) highlighted a lack of research exploring technologies to support leisure activities for people with dementia. Similarly, examining research relating to the use of touchscreen technology by people with dementia, Joddrell and Astell (2016) found that few studies focused on using such technology for entertainment or leisure purposes and recommended this as an avenue for further work, given the usability of touchscreen technology by people with dementia has been established. A scoping review of the use of technology for arts-based activities in older adults living with mild cognitive impairment or dementia conducted by MacRitchie et al. (2022) provides an update on research in this area highlighting devices and applications designed to enable digital music listening and making, art viewing and making, film-making and storytelling. Of relevance to the current review is a study conducted by Tyack et al. (2017) which involved 12 dyads of people with dementia and their carer being asked to use a specially designed application to view a selection of images of works of art sourced from three London museums on a tablet at least five times over two weeks. Qualitative findings from the study highlighted the positive effect that this activity had on giving couples a new shared activity providing prompts for conversation and reflection (Tyack et al., 2017). In addition, Presti (2021) described virtual tours of museum and galleries for older people available via the Google Arts and Culture platform, but cautions that not all live streaming sources and digital museum tours may be suitable for people with dementia.

A systematic review of touchscreen interventions for people with dementia by Tyack and Camic (2017) found that they may confer a wide range of benefits relating to mood, mental health, social engagement, sense of achievement and quality of social interactions. In particular, the review highlighted the potential for art gallery-based interventions, such as those described by Camic et al. (2014), to be adapted to delivery on tablets/touchscreens used at home (Tyack & Camic, 2017).

One of the potential advantages of being able to deliver art interventions online is that participants are not required to attend the gallery in-person, thus extending the reach of such programs to people with dementia who may have mobility or transport access issues or be living in residential aged care or even hospital settings (Hung et al., 2018, 2019, 2021; Stewart et al., 2022). A recent review by Sweeney et al. (2021) on the use of everyday technologies to enhance wellbeing and enjoyment for people living with dementia, found that having a carer able to offer an appropriate level of assistance to the person with dementia to use technology was associated with increased engagement with technology and that shared experiences of using technology with other people with dementia in a group setting helped increase laughter, conversation and enjoyment. However, as people have generally become increasingly familiar with technology during the pandemic due to increased availability, improvements in usability, familiarity, and accessibility, this may represent a confounding variable in studies conducted before the pandemic.

### COVID-19 as a catalyst for increased technology use by older people

In the final theme, seven articles included information on increasing technology use by older people due to the COVID-19 pandemic (Australian Communications & Media Authority, 2021; Brandão et al., 2022; Dowson & Schneider, 2021; Haase et al., 2021; Joddrell & Smith, 2019; Keisari et al., 2021; Lee, et al., 2021). Low levels of digital literacy among older people have traditionally been regarded as barriers to participation. However, Joddrell and Smith (2019) have argued that in the future, the increasing use of everyday technologies such as mobile phones, smartphones, and tablets across the generations means that increasing numbers of people who are diagnosed with dementia will already be familiar with the use of such technologies (Joddrell & Smith, 2019). The changes brought about by COVID-19 may mean that the future has begun to arrive more quickly than was previously expected.

The COVID-19 pandemic served as a catalyst for increasing digital literacy among older adults, with many who had not previously engaged in online video conferencing and social activities starting to engage with digital telecommunications in response to the movement restrictions imposed during 2020 and 2021 (Haase et al., 2021). A report by the Australian Communications and Media Authority (ACMA) in 2021, found that older people increased their online activities during the COVID-19 pandemic, particularly for communication and entertainment. In Australia, 93% of older people have internet access in their homes (as of June 2020); an increase from 68% in 2016 (Australian Communications & Media Authority, 2021). More specifically, during the COVID-19 pandemic, 34% of older Australians started video conferencing and 41% increased their use of such platforms. These significant increases in a relatively short period of time demonstrate the potential for online art activities to reach a large portion of the older population, including people with dementia.

One study examining the delivery of dementia-inclusive online singing groups in Ireland during COVID-19 identified barriers to participation such as lack of access to electronic devices, Wi-Fi, strong internet connections, and/or technical support (Lee et al., 2021). While some participants with dementia were able to independently manage their own participation, others relied on family members or community health workers to assist them to get set up to participate. The program facilitators described a duality of experience in relation to the ways in which online delivery affected access to their programs, while some people were unable to participate, others who had not previously been able to access onsite programs due to their geographic location, health, transport or mobility issues, were now able to participate (Lee et al., 2021). A study by Keisari et al. (2021) evaluated an online creative digital photo collage intervention for older community-dwelling people in Italy and Israel. Thematic analysis revealed that the digital photographs facilitated positive and safe interactions with practitioners, enabling them to process and share life experiences via Zoom in 1.5-h, twice-weekly sessions, during government lockdowns (Keisari et al., 2021). Some participants needed assistance with the technology, while others embraced it by contacting practitioners via other online mediums to share personal thoughts, photographs, and ideas they wanted to incorporate into future sessions.

A scoping review of literature relating to online singing groups for people with dementia (Dowson & Schneider, 2021) completed prior to the COVID-19 pandemic highlighted the potential of such activities to provide digital engagement opportunities for people with dementia. Another study by Brandão et al. (2022) examined the delivery of intergenerational online group activities (dancing, clowning, cooking conversation and storytelling) for vulnerable older people in Brazil, identifying a broader range of challenges to successful participation including hearing difficulties, speech intelligibility, activating microphones and cameras and positioning of electronic devices (and cameras) to capture participant’s face and gestures. Despite these challenges, the researchers highlighted the potential for this and similar remote interventions to help meet the needs of people with dementia for social engagement, enjoyment and creative expression (Brandão et al., 2022).

### Museums and galleries go online – emerging practice

A general internet search showed that many galleries moved to online delivery during COVID-19 shutdowns. Transitioning to online delivery of gallery-facilitated art activities for people with dementia was mainly considered experimental in 2020 (Boag et al., 2022). Cutler (2020, p7) describes that many arts organisations had spent the previous decade or more developing art access programs for older adults, including people with dementia, which “meant that arts organisations confronted the pandemic from a position of strength when it came to working with older people.” Accordingly, many galleries were committed to continuing to provide access to their art conversation or art-making activities for people with dementia. Following the experiences of galleries in other parts of the world that were subject to COVID-19 shutdowns early in 2020, the National Gallery of Australia (NGA) which has run an Art and Dementia program since 2007, trialled online delivery of their art conversations with a small group of participations via Zoom in 2020 during the first lockdown in Canberra and were able to roll out a full program of online sessions in 2021 during a second lockdown (Boag et al., 2022). This move to online delivery was supported by existing relationships between the program facilitators and participants as well as familiarity with the program content, so whilst the mode of delivery changed participants were familiar with other elements of the program.

An action research project involving seven Edinburgh-based cultural and heritage organisations (including the National Galleries of Scotland and Museums & Galleries Edinburgh) conducted in 2021 by Stewart et al. (2022) sought to identify the elements for successful delivery of online events for people with dementia and their caregivers. Stewart et al. (2022) made several recommendations based on qualitative research findings relating to outreach and engagement, event delivery and event longevity including:

 • the need for advanced planning and pre-event interactions between session facilitators and participants to build digital confidence and event familiarity;
 • the use of Zoom as the preferred platform for video conferencing,
 • a preference for mid-morning start time, session duration of no longer than 60 min and a small number of attendees (number not quantified)
 • staff or others involved in delivery of the events should have up to date dementia training
 • participants should be provided with opportunities to provide feedback on events.

Other video conferencing software used by organisations to deliver online sessions include Microsoft Teams (Stewart et al., 2022) and Google Meet (Cutler, 2020).

While art galleries around the world initiated online programs for people with dementia in response to the restrictions imposed by the COVID-19 pandemic, many are continuing to offer online sessions in addition to their onsite programs. In the United States programs such as “Meet Me at MoMA” (Museum of Modern Art, nd), “Arts & Minds”, rebranded as Arts & Minds @home (English) and *en Casa* (Spanish) (Arts & Minds, nd; Halpin-Healy, 2017; Halpin-Healy & Noble, 2020), “See Me at the Smithsonian” (Access Smithsonian, nd), “Met Escapes” at the Metropolitan Museum of Art (The Met, nd), “*here:now*” at the Frye Art Museum (Frye Art Museum, nd) and “Reflections: Alzheimer’s Tours” at the Nasher Museum of Art at Duke University (Nasher Museum at Duke University, nd), also transitioned to online delivery during the pandemic and are continuing to offer online sessions.

The continuation of online art activities for people with dementia is also evident in an online events database published by the Arts4Dementia charity which works with galleries and museums throughout the United Kingdom (Arts4Dementia, nd). The Arts4Dementia events database enables galleries, museums and other arts organisations to list their events and includes information about both in-person and online programs. For example, the Manchester Art Gallery offers fortnightly Art bites sessions online for groups of up to 20 people.

## Discussion

The aim of the review was to identify potential benefits, opportunities and challenges to implementing online gallery-facilitated art activities for people with dementia. One of the primary benefits of implementing online delivery of gallery-facilitated art conversations and art-making groups is the potential for galleries to reach new audiences, particularly people with dementia living in regional, rural or remote areas where access to dementia support services and art galleries may be limited. This mode of delivery also enables galleries to extend their programs to people with dementia who may have difficulty or be restricted from leaving home due to illness, mobility issues or limited access to transport. It may also provide increased access to art for people with dementia who live in residential aged care homes, provided residents at such homes have access to the necessary ICT equipment and related support (Chu et al., 2021). Cutler (2020) observed that in the UK only a minority of residential aged care homes engage with arts organisations that deliver these types of programs and due to increased demands on staff during the pandemic (continuing in many instances through until the present time), the capacity of staff to prioritise facilitation of residents’ involvement in these engagement activities is limited.

While participants in art conversation sessions are not required to do any pre-planning other than logging in to the video conference at the appropriate time, participation in online art-making activities requires some pre-planning in terms of materials and supplies needed in order to ensure that online participants benefit from the failure-free format applied in gallery-based activities (Shoesmith et al., 2021). For example, a program in the United Kingdom used a weekly mail delivery service during COVID-19, sending art boxes to 33 people with dementia for eleven months during the pandemic (Armstrong et al., 2022). Other programs provide a list of supplies before connecting online. In addition, providing appropriate levels of technical support will also be important to encourage sustained involvement in sessions. Other potential challenges, as identified by Brandão et al. (2022), relate to the need for the facilitator to identify and address any access challenges that participants may have, such as hearing difficulties or unfamiliarity with video conferencing equipment, however strategies such as pre-session check-ins with participants could be used to address technological issues.

Further challenges to achieving successful engagement are common to both onsite and online programs, such as group dynamics and individual capacity to participate. Shoesmith et al. (2021) found based on qualitative interviews with participants, the success of visual art interventions was attributed to the presence of a supportive facilitator. In particular, people with dementia described that it was important that they could contribute to the discussions at their own pace without feeling ‘pushed’ to contribute and they referred to the encouragement and support provided by the facilitator more than other elements of arts interventions (Shoesmith et al., 2021). Based on research by Sweeney et al. (2021) relating to the important role that carers play in providing an appropriate level of assistance to the person with dementia to use technology it is likely that successful participation in online groups will in some circumstances require involvement of carers to assist participants to access relevant video conferencing tools and potentially prompting and assisting participants to log in to scheduled sessions. Research is needed to understand how the success of online programs can be affected by carers with different levels of familiarity with using technology and carer burden as the carer may need to invest their time in setting up and monitoring online activities.

A systematic review of online singing interventions found barriers and enablers to delivery and use of technology was not specific to people with dementia and carers, and recommendations for online engagement used for other groups apply in the same ways (Dowson & Schneider, 2021). Recommendations included maximising ease of use of the online interface, providing tips and instructions on how to promote engagement, and improving technology and software to preserve a sense of togetherness without video or audio latency. Manera et al. (2022) provide a strengths, weaknesses, opportunities and threats analysis of the use of online (remote) workshops. A comparison of potential advantages and barriers between online and onsite delivery is presented in Table 2.

**Table 2.** Comparison of advantages and disadvantages between onsite and online delivery.

	Onsite		Online	
	Disadvantages	Advantages	Disadvantages	Advantages
Transport	Participant may require assistance to get to/from gallery or museum.	Participant may have access to group transport to get to/from gallery. Opportunity for participant to go out.	Participant does not have opportunity to travel (go out) to participate in activity.	No transport required, participant can attend from anywhere.
Physical activity/Mobility	Participant may require mobility assistance at gallery.	Participant has access to mobility assistance at gallery and gets incidental physical activity through participation in gallery visit.	Participant misses out on incidental physical activity as there is no need to go out.	Participants with mobility or physical disability may find it easier to participate online.
Availability	Gallery staff and scheduling may be limited by other onsite programs, venue capacity, and crowds.	Art and dementia programs part of broader gallery access activities, providing inclusive approach.	Smaller galleries may have less capacity to deliver online access programs.	Gallery staff may deliver programs from various locations and are not required to be physically present at gallery.
Geography/Location	Only people who live near gallery can participate (often not available for people in regional or remote areas).	May be available in local area particularly if living in a major city.	Location of participant may limit availability of reliable internet connection required for participation.	Participants may engage while living in regional or remote areas (or even another state or country) to where gallery is located.
Accessibility	May be challenging for people with mobility issues to attend.	Galleries set up to provide accessibility support for participants.	Participants need reliable internet connection, computer equipment, and either some knowledge of digital technology or support to connect.	May be easier for people in residential aged care homes to participate.
Equipment	N/A	Gallery provides art-making materials and equipment.	Participants may not have access to suitable computer equipment (laptop, tablet or desktop computer) and may need to provide own art-making materials.	Participants can use existing computer equipment to participate in new activity.
Social engagement	May be challenging for participants to engage during busy times in the gallery.	Participants have face-to-face interactions and opportunity for before/after social interactions.	Participants may find interacting onscreen is not as satisfying as in-person interactions. More difficult to have 1:1 conversations with other participants.	Participants can engage socially via video conferencing.
Setting	Participants may find gallery setting too busy or noisy or unfamiliar/disorienting.	Participants get to experience pleasure of being in “special and valued” gallery space.	Participants do not experience pleasure of attending gallery space.	Participants may be more comfortable in familiar/home setting.
Art	Participants only have access to works of art that are on display in gallery.	Participants see original work of art and can view work of art in context of exhibition. Participants view works of art in 3D and from multiple angles.	Participants can only view works of art in 2D (onscreen). Participants may only be able to view work of art from one angle.	Participants have opportunity to view works of art from digital collection of gallery (not limited to what is on display).

### Future directions

This review explores a novel area of gallery-based art activities for people with dementia. Given the scarcity of topic-specific literature, it acts as a starting point for future research on this topic. There is a need to compare different types of online programs and following Shoesmith et al. (2020), evaluate interventions which incorporate the elements required to deliver an effective online visual art intervention and support successful online participation for people with dementia. These elements include the dose, interesting and enjoyable content, and a structure which typically involves art-viewing and discussion, then art-making. It would also be instructive to consider the barriers to online participation by examining the experiences of those who withdrew from or chose not to participate in online programs during the COVID-19 lockdowns due to technological issues, or lack of interest or support. As weekly attendance is the current standard, yet the length of intervention does not appear to influence the ability of arts activities to enhance wellbeing (Shoesmith et al., 2020), research focused on sustainable attendance via online delivery, or indeed flexible delivery models, should be considered. In addition, the benefits of being able to provide access to these types of art-based interventions for people with dementia living outside major cities, in regional or remote areas where access to dementia support services may be limited, might also provide insights into the potential for other dementia support services and activities to move to an online format. The provision of online art activities to people with dementia living in residential aged care and hospital settings, building on the work of Hung et al. (2018) is also another potential topic for further research.

To identify whether the types of benefits described in the literature relating to onsite programs are evident in online programs, future research should seek to compare each type of intervention. Despite the continuing presence of COVID-19 at the time of writing, a resumption of normal activities is occurring worldwide. On balance, it appears better to have online art programs available to people with dementia and carers who would otherwise not be able to access them, such as people in remote and rural areas and people living in residential aged care. With current technologies, there will be different levels of social engagement between online and onsite programming. However, several difficult to measure or overlooked components of onsite programming exist. For example, the act of a person with dementia attending museums and galleries in-person can have multiple benefits such as a reduction in sedentary behaviour and increased steps and time spent outside, and a sense of purpose or self-worth possibly related to getting themselves dressed and ready for the outing. Therefore, future research comparing a broad range of online and onsite delivery outcomes are needed. In addition, exploration of benefits to carers and whether their participation has unexpected benefits or adverse effects (Kinsey et al., 2022), participation online in small groups of people with dementia versus alone, and the feasibility of delivery in residential aged care and hospital settings are areas of interest which could contribute to greater adoption of online art activity program delivery. Meaningful activities and experiences for people with dementia can provide an opportunity for carer respite, and the opportunity for respite will be different between online and onsite programs relative to the assistance required to facilitate participation. For both online and onsite programs to be sustainable, it is essential for strong partnerships between museums and galleries, clinicians, dementia advocacy groups, and researchers to be in place to support continuous delivery.

### Limitations

The present review is limited to literature published in English. While the search strategy was far-reaching and included grey literature and citation searching, given the multi-disciplinary nature of the literature relating to this topic, relevant articles and reports may have been missed. Furthermore, given the growing amount of research into online interventions in response to the COVID-19 pandemic, it is anticipated that a more comprehensive review or systematic review in the coming years will add to the literature surrounding the online delivery of gallery-facilitated art interventions for people with dementia and carers. Few articles described their cohorts in detail; therefore, we were unable to evaluate whether people with different types of dementia may respond variably to online delivery. As people with types of dementia which lead to visual challenges or language impairments may have difficulty engaging in online environments, future research should investigate the effects of dementia type on potential benefits of online art activities.

## Conclusion

This narrative review has identified the potential for art galleries to expand the reach of their existing programs for people with dementia through online delivery of art conversations and art-making activities. Key benefits of using online delivery relate to providing access to gallery-facilitated art activities for people with dementia in regional and remote areas who otherwise could not participate in such programs; as well as providing new opportunities for online social interaction and engagement in art activities for people with dementia. There is a significant need for research to examine the efficacy of this mode of program delivery and to understand enabling strategies used by facilitators of such programs to support successful participation and social engagement among participants. The main barriers to participation in gallery-facilitated online art activities relate to digital literacy and digital exclusion, primarily having access to necessary equipment and technical support in order to participate in online activities. Given the increasing evidence relating to the positive impact of participation in gallery-facilitated art activities for people with dementia, online delivery has exciting potential to significantly extend the reach of such programs to a larger number of people with dementia.
